# Vaccination for meningococcal disease: Number 9 – September 2022

**DOI:** 10.1055/s-0042-1757755

**Published:** 2022-10-10

**Authors:** Cecilia Maria Roteli-Martins, Nilma Antas Neves, Valentino Antonio Magno, Renato Kfouri

**Affiliations:** Centro Universitário Faculdade de Medicina do ABC, Santo André, SP Brazil.; Universidade Federal da Bahia, Salvador, BA, Brazil.; Universidade Federal do Rio Grande do Sul, Porto Alegre, RS, Brazil.; Centro de Imunização Santa Joana, São Paulo, SP, Brazil.; Centro de Imunização ProMatre Paulista, São Paulo, SP, Brazil.

## Key points

- Update gynecologists and obstetricians on the importance and need for vaccination against meningococcal meningitis, raising awareness of the risk of invasive meningococcal disease in adolescents and young adults, with positioning of the National Commission Specialized in Vaccines of the Brazilian Federation of Gynecology and Obstetrics Associations (Febrasgo) about the subject.
- Show that meningococcal infection can result in serious diseases, such as meningitis and meningococcemia, and in Brazil, at least 2 out of 10 people with invasive meningococcal disease die from it. Furthermore, about 20% of survivors have permanent sequelae.
- Present the different vaccines available and their characteristics, as well as the official recommendations in Brazil.
- Emphasize that the protective efficacy of vaccines for ACWY serogroups declines over time when administered in childhood, and booster doses in adolescents and young adults are important.
- Inform that in the Brazilian National Immunization Program (PNI), the meningococcal ACWY conjugate vaccine is routinely available to 11- and 12-year-old adolescents.
- Inform that meningococcal ACWY and B vaccines are available for different age groups in private vaccination clinics, as indicated in the package insert and recommended by scientific societies.
- Present the best scientific evidence of vaccination against meningococcal meningitis in women and contribute to an up-to-date clinical practice.
- Emphasize the importance of gynecologists discussing vaccination against meningococcal disease with adolescents, parents and legal guardians.

## Recommendations

- Reinforce the importance of meningococcal vaccination in adolescents and young adults given the high risk of this population contracting meningococcal disease. This is a very serious disease with serious outcomes; even death can occur in just a few hours. Licensed conjugate vaccines can also eliminate the asymptomatic carrier state, common in this age group.
- Recommend meningococcal vaccination in immunosuppressed women of any age given their higher risk of developing meningococcal disease, including severe forms
- Endorse that, in Brazil, as well as in other countries where they were adopted, meningococcal vaccines reduced the prevalence of the disease, which justifies their use according to the current recommendation presented in the text.
- Demonstrate that meningococcal ACWY and B conjugate vaccines are effective and promoted significant reduction in cases of meningococcal disease in all settings and places where they were introduced. They have an excellent safety profile, with no reports of serious adverse events.
- Emphasize that quadrivalent meningococcal conjugate vaccines (types A, C, W, Y) and protein vaccines against serogroup B, due to their greater spectrum of protection, should be considered as the best options for immunization of adolescents and adult women, when available.
- Determine that in all age groups, if using the ACWY and B vaccines is not possible, the monovalent C meningococcal vaccine can be used.
- Determine that in situations of bacterial meningitis outbreak, block vaccination is used after identifying the responsible serogroup and following the technical standards recommended by the National Immunization Program of the Ministry of Health.
- Reinforce that the protective efficacy of ACWY vaccines declines over time, especially when administered to young children. As high antibody titers are essential for adequate protection, the need for booster doses is evident to maintain these high levels in adolescents and young adults.
- Demonstrate that considering all age groups, serogroup B was responsible for 36% of serogrouped cases of invasive meningococcal disease in Brazil in 2020, and recommend vaccination for this serogroup in adolescents and young adults.

## Background

Invasive meningococcal disease is caused by Neisseria meningitidis (meningococcus), a Gram-negative, encapsulated diplococcus that can cause serious infections such as meningitis and septicemia. Meningococcus is the main causative agent of bacterial meningitis in Brazil. Although 12 serogroups have been identified, almost all invasive meningococcal diseases are caused by A, B, C, W and Y serogroups. 1

Infections usually have an abrupt onset and rapidly progress from nonspecific flu-like symptoms to the classic forms of meningitis and septicemia within eight hours of the onset of symptoms. This condition can progress to worsening and even death within 24 hours. Severity and death rates are high, particularly in adolescents and young adults, with a high rate in older adults as well. 2

In up to 20% of invasive disease survivors, sequelae such as deafness, cognitive system dysfunction, learning difficulties, amputations and other neurological sequelae are common. The disease also has a negative impact on the quality of life of survivors and their families, as it can cause neurological problems that last for more than 21 months. 3

Of all disease cases in Brazil in 2019, 53.7% were not serogrouped in the laboratory and 46.3% were classified as serogroups B, C, W and Y among all age groups. Among the four main serogroups identified, the highest percentage corresponded to serogroup C (48.6%), followed by serogroup B (40.1%), serogroup W (7.7%) and serogroup Y (3.6%). In 2020, during the COVID-19 pandemic, despite the lower number of N. meningitidis isolates, the percentage contribution of cases of invasive disease by serogroup B remained higher in groups aged under 15 years. 4 5 6

## How is the disease transmitted?

Meningococcal disease is transmitted through contact with an asymptomatic carrier or a sick person, or direct contact with respiratory secretions. Nasopharyngeal colonization is a necessary condition for the development of the disease. Most of the time, the carrier is asymptomatic, even though in some cases bacteria can invade the bloodstream, reaching previously sterile sites, such as the cerebrospinal fluid and the brain. 1 In a systematic review of studies in patients with N. meningitidis, children represent 4.5% of carriers of the bacterium and the rate increases to 23.7% in adolescents around 19 years of age and decreases to 7.8% in adults aged 50 years. 7 The incubation period is often very short, around two days, and the period of transmission persists until the meningococcus disappears from the nasopharyngeal secretions of the patient or carrier, which in general, occurs at 24 hours after starting the specific treatment. 8

## What are the groups at higher risk?

The main risk factors for the transmission of N. meningitidis are associated with the behavior and social habits of adolescents and young adults, such as, for example, attending spaces and living at universities, gathering at parties and events, sharing objects of personal use, utensils, kisses, among others. 1 In an Australian study of students aged 15-18 years, risk factors associated with the asymptomatic carrier state were identified and significantly related to the use of regular cigarettes (odds ratio [OR]: 1.91, 95% confidence interval [CI]: 1.29-2.83) and waterpipe tobacco (OR: 1.82, CI 95%: 1.30-2.54), bar or club goers (OR: 1.54, 95% CI: 1.28-1.86) and intimate kisses (OR: 1.65, CI of 95%: 1.33-2.05). 7 In Embu das Artes, São Paulo, a study showed that about 12.5% of adolescents are carriers of N. meningitidis, with the following serogroup (SG) prevalence: SGC (18.4%), SGB (12.6%), SGY (4.6%), SGW (1.1%) and non-serogrouped (60.9%), showing the importance of the carrier status in a region of Brazil. 9 Note that immunocompromised adolescents, those with underlying chronic diseases, and those with paroxysmal nocturnal hemoglobinuria using eculizumab are at a higher risk for invasive meningococcal disease. 1

## What are the symptoms?

The main signs and symptoms of the disease are fever, intense headache, vomiting, prostration, convulsions, signs of meningeal irritation and hemorrhagic suffusions. Meningococcemia (septicemia) is the most severe clinical form of meningococcal disease, has high case fatality rates and may or may not be associated with meningitis. 10 High case fatality rates, ranging from 10% to 20%, have been reported, and are higher in serogroup W (>30%). Among survivors, permanent sequelae are observed in about 10-20% of cases: limb amputation, deafness, vision loss, memory and learning difficulties, among others. 11 In Brazil, an increase in disease incidence associated with serogroup W was observed recently, which was explained by the higher number of cases in Santa Catarina in years 2017 and 2018. 12

## What vaccines are licensed in Brazil?

Vaccination is considered the most effective way to prevent the disease. A variety of vaccines, polysaccharide and conjugate, have been developed to help protect against meningococcal serogroups A, C, W, and Y. The immune response triggered by infection or vaccine use is predominantly serogroup-specific. 13 Limitations of polysaccharide vaccines include hyporesponsiveness and lack of impact on the carrier state, and conjugate vaccines help to solve some limitations of polysaccharide vaccines, with better immune response in pre-teens, adolescents and adults. Conjugate vaccines, unlike polysaccharides, also induce immunological memory (booster effect), contributing to reduce carriers and to indirect protection. 14 Currently, in Brazil, there are licensed conjugate vaccines available against four serogroups – A, C, W and Y – in monovalent (C) or quadrivalent (ACWY) formulations. Whenever possible, in any age group, the use of quadrivalent ACWY vaccines is preferable given their greater protection spectrum. In all ages, if use of the ACWY vaccine is impossible, the meningococcal monovalent C (MenC) vaccine should be used. 15 After introduction of the MenC vaccine in the National Immunization Program, the incidence rate of meningococcal disease by serogroup C reduced significantly, with a proportional increase in cases related to serogroup B. Meningococcal ACWY and B vaccines are available for different age groups in private vaccination clinics, as indicated in the package insert and recommended by scientific societies. 16 17 Meningococcal C conjugate vaccine was the first conjugate vaccine approved against meningococcus. Its introduction into public programs showed a marked reduction in the number of cases of the disease among those vaccinated. Countries that included adolescents in their vaccination programs obtained even more expressive results in indirect protection, as most asymptomatic carriers of the bacterium are concentrated in this age group. In adolescents and adults, the regimen is a single dose. The Brazilian Immunization (SBIm) and Pediatrics (SBP) Societies and the Brazilian Federation of Gynecology and Obstetrics Associations (Febrasgo) recommend vaccination for all individuals up to 20 years of age on a routine basis. Above this age, the vaccine can also be used, especially in groups at greater risk for acquiring the disease or in situations of epidemics or travel to places of greater risk. For adolescents, adults or older adults with indication, a single dose is recommended. 16 17 18 19 Meningococcal ACWY conjugate vaccine includes serogroups A, C, W and Y. It expands the protection spectrum in relation to the monovalent C vaccine and has been recommended preferably in the child and adolescent calendar by scientific societies. In adults, it is also reserved for the same already described situations related to the monovalent C vaccine. 17 18 Recombinant meningococcal B vaccine: there are two vaccines licensed in Brazil, and their use is recommended for children, adolescents and young adults. In clinical trials, meningococcal B vaccines have demonstrated a robust immune response in adolescents lasting up to 7.5 years and an acceptable safety profile. Recent evidence from the routine use of meningococcal B vaccine has demonstrated a reduction in cases of invasive meningococcal disease by this serogroup among those vaccinated aged between 2 months and 20 years, with a consistent safety profile with that seen in clinical trials. 7

## What is the safety and adverse effects?

The most common adverse events are local pain, redness and swelling at the site. Systemic events are usually mild and of short duration, such as fever, malaise, drowsiness, vomiting, headache and body pain. 13 Especially in children, the meningococcal B vaccine is usually more reactogenic, leading to febrile conditions in up to 50% of cases. 20

## What is the current recommendation?

The meningococcal C vaccine was included in the National Immunization Program in 2010 for children aged 3 to 24 months. Subsequent records show a significant drop in the number of cases and deaths in the age groups in which the vaccine was used. Thus, as of 2017, the meningococcal C conjugate vaccine was made available also for adolescents aged 11 and 12 years to control a possible decline in immunity observed over time. In 2020, the Ministry of Health included the meningococcal ACWY conjugate vaccine for adolescents aged 11-12 years as a booster dose. 21 Societies, including the Brazilian Federation of Gynecology and Obstetrics Associations (Febrasgo), also recommend the preferential use of MenACWY vaccines for adolescents in a two-dose regimen at a five-year interval in between whenever possible. When administration to adults is justified, the recommendation is a single dose. 17 18 The vaccine against serogroup C for adults is available only at Reference Centers for Special Immunobiologicals (Portuguese acronym: CRIEs) for immunocompromised patients and those at risk for acquiring the disease, in the two-dose regimen at a five-year interval in between. In cases of epidemics, consider vaccinating women aged > 60 years. 22 Meningococcal B and quadrivalent conjugate vaccines (types A, C, W and Y) should be considered the best options for immunizing adolescents and adult women. Vaccination with a single dose of ACWY vaccine and two doses of meningococcal B at a 1-month interval in between is recommended. Block vaccination is indicated in situations where there is an outbreak of meningococcal disease provided that the responsible serogroup is known. The vaccination strategy will be defined by the National Immunization Program of the Ministry of Health. 18 23 The meningococcal ACWY conjugate vaccine is available at Reference Centers for Special Immunobiologicals for patients with paroxysmal nocturnal hemoglobinuria who will start treatment with eculizumab and for those with underlying diseases, “in order to update the vaccination calendar”. 24 25

## Final considerations

Meningococcal infection can result in serious illnesses such as meningitis and meningococcemia. Infections usually have an abrupt onset and can progress rapidly, with serious outcomes. Habits of adolescents and young adults make them the main carriers and transmitters of meningococcus in the community. The National Commission Specialized in Vaccines of the Brazilian Federation of Gynecology and Obstetrics Associations (Febrasgo) recommends vaccination with ACWY conjugate vaccines for all pre-teens and adolescents in a two-dose regimen at a five-year interval in between. A booster dose given at 16 years of age gives adolescents ongoing protection during the ages they are at higher risk. Whenever possible, adolescents and young adults should also receive the meningococcal B vaccine in a two-dose regimen at an interval of 1-2 months in between. The National Commission Specialized in Vaccines of the Brazilian Federation of Gynecology and Obstetrics Associations (Febrasgo) emphasizes that multivalent vaccines (ACWY) and those against meningococcal B are indicated for all adolescents up to 20 years of age and for women in the risk group for meningococcal disease.

National Commission Specialized in Vaccines of the Brazilian Federation of Gynecology and Obstetrics Associations (Febrasgo)

President:

Cecilia Maria Roteli Martins

Vice-president:

Nilma Antas Neves

Secretary:

Susana Cristina Aidé Viviani Fialho

Members:

André Luís Ferreira Santos

Angelina Farias Maia

Fabíola Zoppas Fridman

Giuliane Jesus Lajos

Isabella de Assis Martins Ballalai

Juarez Cunha

Júlio Cesar Teixeira

Manoel Afonso Guimarães Gonçalves

Marcia Marly Winck Yamamoto de Medeiros

Renata Robial

Renato de Ávila Kfouri

Valentino Antonio Magno
